# Clinical characteristics and prognosis of ovarian clear cell carcinoma: a 10-year retrospective study

**DOI:** 10.1186/s12885-021-08061-7

**Published:** 2021-03-25

**Authors:** Chenchen Zhu, Jing Zhu, Lili Qian, Hanyuan Liu, Zhen Shen, Dabao Wu, Weidong Zhao, Weihua Xiao, Ying Zhou

**Affiliations:** 1 Department of Obstetrics and Gynecology, Anhui Provincial Hospital, Anhui Medical University, Hefei, 230001 China; 2 Department of Obstetrics and Gynecology, The First Affiliated Hospital of USTC, Division of Life Sciences and Medicine, University of Science and Technology of China, Hefei, 230001 Anhui China; 3 Division of Molecular Medicine, Hefei National Laboratory for Physical Sciences at Microscale, The CAS Key Laboratory of Innate Immunity and Chronic Disease, School of Life Sciences, University of Science and Technology of China, Hefei, China; 4 Institute of Immunology, University of Science and Technology of China, Hefei, China

**Keywords:** Ovarian clear cell carcinoma, Biomarker, Survival, Prognostic factor

## Abstract

**Background:**

Ovarian clear cell carcinoma (OCCC) is a special pathological type of epithelial ovarian carcinoma (EOC). We conducted this research to investigate the clinical characteristics and outcomes of OCCC and to provide additional supporting evidence to aid in the clinical diagnosis and management.

**Methods:**

This was a retrospective study investigating the clinical characteristics and survival outcomes of 86 patients with OCCC treated at our center between January 2010 and March 2020. Survival analysis was also performed on 179 patients with OCCC obtained from the Surveillance, Epidemiology and End Results (SEER) cancer registry database.

**Results:**

The median age of participants was 49.21 ± 9.91 years old, and 74.42% of them were diagnosed at early stage. The median CA125 level was 601.48 IU/mL, while 19.77% of the patients had normal CA125 levels. Sixteen patients (18.60%) had co-existing endometriosis and 8 patients (9.3%) developed venous thromboembolism (VTE). There were 5 patients received suboptimal cytoreduction. Sixty-six patients (76.74%) underwent lymphadenectomy, and only 3 (4.55%) patients had positive lymph nodes. Patients diagnosed at an early stage had higher 3-year overall survival (OS) and progression-free survival (PFS) rates than those with advanced stage OCCC. CA19–9 (*P* = 0.025) and ascites (*P* = 0.001) were significantly associated with OS, while HE4 (*P* = 0.027) and ascites (*P* = 0.001) were significantly associated with PFS. Analysis of data from the SEER database showed that positive lymph nodes is also an independent prognostic factor for OS (*P* = 0.001).

**Conclusions:**

OCCC often presents at an early stage and young age with a mildly elevated CA125. CA19–9, HE4, massive ascites, and positive lymph node are independent prognostic factors.

**Supplementary Information:**

The online version contains supplementary material available at 10.1186/s12885-021-08061-7.

## Background

Epithelial ovarian carcinoma (EOC) is the seventh most commonly diagnosed cancer among women worldwide, and carries the highest mortality rate of all gynecological cancers [1, 2]. Ovarian clear cell carcinoma (OCCC) is a subtype of EOC with differing prevalence depending on geographical location. OCCC accounts for 5–10% of all EOC in North America and 12% in western countries, but appears to have a higher prevalence in East Asia, accounting for 25–30% and 10.3–11.6% of all EOC in Japan and Korea, respectively [3–5].

There are several reproductive and hormonal risk factors linked to an increased risk of developing OCCC, such as early menarche, late menopause, low use of oral contraceptives, and low pregnancy rate [6]. In addition, endometriosis is recognized as a precancerous lesion of OCCC, as women with endometriosis have a 3-fold increased risk of developing OCCC compared with women without endometriosis [7]. Patients with OCCC tend to be diagnosed at younger age and earlier stage, and occasionally are found to have thromboembolic complications [8], usually with a mild-to-moderate elevation of serum CA125. The conventional tumor marker CA125 used for detection of high-grade serous carcinoma (HGSC) is a poor marker for OCCC, elevated in only 57.6% of OCCC cases with a high false-negative rate [9]. Therefore, there is a need for specific serological biomarkers for OCCC. Immunohistochemically, OCCC is usually positive for hepatocyte nuclear factor 1β (HNF1β) and negative for estrogen receptor (ER), progesterone receptor (PR), and Wilms Tumor 1 (WT-1) in more than 95% of the cases [10]. OCCC shows little association with family history, and BRCA1 and BRCA2 germline mutations are rare in OCCC, however, somatic mutations of phosphatidylinositol-4,5-bisphosphate 3-kinase catalytic subunit alpha (PIK3CA) and AT-rich interactive domain-containing protein 1A (ARID1A) are present in 20–51% and 40–57% respectively [11, 12].

Standard surgical staging procedure or optimal cytoreduction, followed by systemic chemotherapy, is recommended as the primary treatment for patients with OCCC. However, the response rate of platinum-based chemotherapy is only 20 to 50% for OCCC, therefore there is a need for further research into more effective therapies [13]. Due to this inherent chemoresistance, the prognosis of patients with OCCC is extremely poor, especially at advanced stage. Suboptimal cytoreduction, lymph node (LN) metastasis, and occurrence of VTE are also prognostic predictors of poor outcome [13, 14].

Therefore, the purpose of our research is to assess the clinical characteristics and outcomes of patients with OCCC, and to provide additional supporting evidence to aid in the clinical diagnosis and management of OCCC.

## Materials and methods

### Patients

This is a retrospective study of 86 patients diagnosed with primary ovarian clear cell carcinoma between January 2010 and March 2020 at The First Affiliated Hospital of University of Science and Technology of China (USTC). Patients with histologically confirmed OCCC with only pure clear cell histology by pathologists who had undergone complete surgical staging or cytoreductive surgery with adjuvant chemotherapy as the primary treatment were included. Patients were excluded from this study if they received neoadjuvant chemotherapy, had insufficient data, or were lost to follow-up within 1 month of surgery. Patient information, including demographic and pathological characteristics, pre-operative biomarkers, surgical procedure, chemotherapy, and disease status at last contact, was collected from medical records and evaluated. Patient records and information were anonymized prior to analysis; thus, consent was not required. This study was approved by the ethics committees of The First Affiliated Hospital of USTC and was conducted in accordance with the Helsinki Declaration.

### Treatment and follow-up

The predominant primary surgical procedure was total hysterectomy, bilateral salpingo-oophorectomy, lymphadenectomy, and omentectomy, with peritoneal biopsies from multiple random sites. Twenty-five patients with a pelvic mass had previously undergone surgical procedures including oophorocystectomy, unilateral/bilateral ovariosalpingectomy ± total hysterectomy, and were diagnosed with OCCC according to the pathological results from our or other hospitals and subsequently received additional staging or cytoreductive surgery in our hospital. The other 61 patients were diagnosed and treated within our hospital. Firstline adjuvant chemotherapy was combined platinum and taxanes of 3–6 cycles.

After the initial treatment, all patients were closely followed up with clinical examination, including pelvic examination and evaluation of tumor markers at each visit. In addition, ultrasound, computed tomography (CT), magnetic resonance imaging (MRI), and/or positron emission tomography-CT (PET-CT) scans were performed when necessary. Where follow-up information was not available from patient records, patients were contacted directly by telephone to obtain the relevant information. Recurrence was defined as histologic evidence by tumor biopsy or fine-needle biopsy and/or the appearance of new lesions on imaging. Survival data were last collected on 31 April 2020.

### Clinical data collection

The following information was collected from the medical records of eligible patients: age, body mass index (BMI), results of genetic tests, presence of endometriosis, history of thromboembolism, stage, comorbidities, American Society of Anesthesiologists class (ASA), stage, preoperative serum laboratory test values, surgical procedures performed, presence of ascites, size of residual tumor, number of LN removed, presence of LN metastasis, pathologic results, length of hospital stay, chemotherapy regimen, length of follow-up, recurrence and survival status.

Fasting venous blood samples were collected from all patients on the morning prior to their planned surgery. Electrochemiluminescence immunoassay (ELICA) was performed on all samples using the Cobas E601 analyzer (Roche Diagnostics) to measure the levels of CA125, HE4, and CA19–9 For patients who had undergone primary surgery at other institutes, the results of serum analysis were collected from their medical notes. All tumors were staged according to the 2014 International Federation of Gynecology and Obstetrics (FIGO) staging system. In patients treated prior to 2014, the stage of disease was classified retrospectively on the basis of surgical and pathological assessment. Optimal cytoreduction was defined as the maximal diameter of the residual tumor ≤1 cm following surgery. Progression-free survival (PFS) was defined as the time from initial surgical staging or cytoreductive surgery to the date of disease progression or recurrence, and overall survival (OS) was defined as the time from surgical staging or cytoreductive surgery to the date of death, or to the last follow-up date, if still alive.

The histological cell types were determined according to the World Health Organization (WHO) criteria, and the diagnosis was conducted by at least two pathologists. Pathological slides of patients who underwent primary surgery at other institutes were obtained for histological reconfirmation. The presence of endometriosis was obtained from pathological reports.

### Data collection from the surveillance, epidemiology and end results (SEER) database

Patients’ data were collected from the latest version of the SEER cancer registry database. There were 849 cases initially identified with a diagnosis of OCCC from 1975 to 2017 with at least 3 years of follow-up available. Exclusion criteria were as follows: incomplete clinical information (621 cases), no surgical procedure performed (8 cases), and the presence of primary malignancy elsewhere (41 cases). Following the selection process, a total of 179 eligible patients were enrolled in the study. Survival analysis was performed using the following demographic and clinicopathological parameters: age, race (white, black, or ‘other’), SEER summary stage (localized, regional, or distant), American Joint Committee on Cancer (AJCC) stage (I, II, III, or IV), number of LN resected (1, 2, 3, or ≥ 4), number of positive LN (1, 2, 3, or ≥ 4), and the presence of distant metastasis.

### Statistic analysis

Statistical analysis was performed using SPSS v.20.0 software (IBM Corp., Armonk, NY, USA). We conducted the power calculation using PASS v11.0 software. Continuous variables were expressed as mean, standard deviation, and range, and categorical variables were expressed as counts and percentages. We constructed the reverse Kaplan-Meier curve by reversing the “censor” and “event” of the standard Kaplan-Meier curve. Comparisons between groups were analyzed using Student’s t-test or Wilcoxon-Mann-Whitney test according to the data distribution for continuous variables or the χ2 or Fisher’s exact test for categorical variables. Univariate and multivariable Cox regression analyses were performed to identify predictors of RFS and OS. Multivariate analysis was performed with all variables with a *p*-value of < 0.1 at univariate analysis. Multivariate logistic regression analysis was also performed with bootstrap in SPSS. A *p*-value of < 0.05 was considered statistically significant, and all *p*-values reported were two-sided.

## Results

### The characteristics of the patients with OCCC

**Fig. 1:**
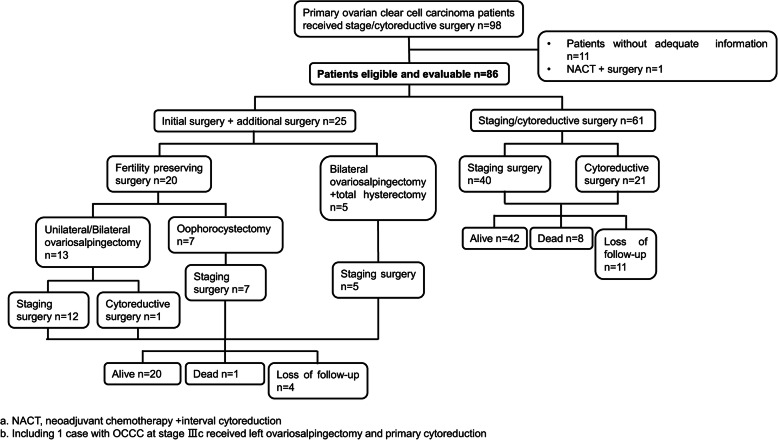
The inclusion process and treatment process of the 86 patients recruited in this study. a. NACT, neoadjuvant chemotherapy + interval cytoreduction; b. Including 1 case with OCCC at stage IIIC received left ovariosalpingectomy and primary cytoreduction

Overall, 86 patients were included in this study who were diagnosed with OCCC between January 2010 and March 2020. The demographic and clinical characteristics of these patients are summarized in Table 1. Twenty-five patients with a pelvic mass had previously undergone surgical procedures including oophorocystectomy, unilateral/bilateral ovariosalpingectomy ± total hysterectomy, and were diagnosed with OCCC in our or other hospitals according to pathological results. Of these patients, 24 of them received additional staging surgery and were diagnosed as early stage, and one patient received cytoreductive surgery and was diagnosed as FIGO IIIC in our hospital. The other 61 patients received primary cytoreductive or staging surgery within our hospital, and detailed information is presented in Fig. 1.

**Table 1:** Baseline clinical characteristics of patients (*n* = 86)

	No	%
**Age (years)**^**a**^	49.21 ± 9.91(25 ~ 70)	
< 40	15	17.44
40 ~ 49	27	31.40
50 ~ 59	33	38.37
≥ 60	11	12.79
**Body mass index (kg/m**^**2**^**)**	22.96 ± 3.01 (16.94 ~ 33.71)	
< 18	2	2.33
18 ~ 23.9	52	60.47
24 ~ 27.9	26	30.23
≥ 28	3	3.49
NA	3	3.49
**FIGO stage**		
I	56	65.12
IA	25	29.07
IB	2	2.33
IC	29	33.72
IC1	13	15.12
IC2	9	10.47
IC3	2	2.33
IC NA	5	5.81
II	3	3.49
IIA	0	0
IIB	3	3.49
III	24	27.91
IIIA	4	4.65
IIIB	1	1.16
IIIC	19	22.09
IV	3	3.49
**BRCA mutation (*n*** **= 15)**		
+	1	6.67
-	13	86.67
Unknown significance mutation	1	6.67
**Endometriosis**	16	18.60
**Thrombosis**	8	9.30
Preoperative	4	4.65
postoperative	4	4.65
**CDC**^**b**^	31	36.05
Hypertension	16	18.60
Diabetes	3	3.49
Heart disease	3	3.49
Cerebral infarction	6	6.98
Other cancer history	4	4.65
Hepatic cancer	1	1.16
Breast cancer	1	1.16
Cervical cancer	1	1.16
Endometrial cancer	1	1.16
Hepatitis	2	2.33
Systemic lupus erythematosus	1	1.16
Rheumatoid arthritis	1	1.16
Bronchial asthma	1	1.16
Hypothyroidism	1	1.16
Moyamoya disease	1	1.16
Tuberculosis	1	1.16
**American Society of Anesthesiologists class (ASA)**		
I	7	8.14
II	39	45.35
III	31	36.05
IV	3	3.49
NA	6	6.98
**Preoperative laboratory test**		
CA125(U/ml) ^a^	601.48 ± 1546.66(9.16 ~ 9035.00)	
Normal (< 35)	17	19.77
35 ~ 99	16	18.60
100 ~ 499	23	26.74
500 ~ 999	4	4.65
≥ 1000	10	11.63
NA	16	18.60
CA19–9(U/ml) ^a^	1575.97 ± 11,885.76(0.60 ~ 96,649.00)	
Normal (< 37)	36	41.86
37 ~ 99	13	15.12
100 ~ 499	14	16.28
≥ 500	2	2.33
NA	21	24.42
HE4 (pM) ^a^	105.37 ± 102.93(14.00 ~ 669.00)	
Normal (< 140)	45	52.33
140 ~ 499	12	13.95
≥ 500	1	1.16
NA	28	32.56
Serum albumin (g/l) ^a^	40.74 ± 4.47(31.10 ~ 51.60)	
< 40	31	36.05
≥ 40	38	44.19
NA	17	19.77
Prealbumin (mg/l) ^a^	194.65 ± 56.29(58.00 ~ 332.00)	
< 170	20	23.26
≥ 170	43	50.00
NA	23	26.74
D-Dimer (ug/ml) ^a^	2.21 ± 3.19(0.03 ~ 12.86)	
< 3.5	47	54.65
≥ 3.5	9	10.47
NA	30	34.88
Ca^2+^(mmol/l) ^a^	2.32 ± 0.25(1.90 ~ 3.50)	
< 2.11	7	8.14
2.11 ~ 2.52	54	62.79
> 2.52	7	8.14
NA	18	20.93

*NA* Not available

^a^Mean ± standard deviation, range

^b^*CDC* Chronic disease comorbidities

The median age at diagnosis was 49.21 ± 9.91 years (range 25–70 years). The majority of patients (64/86, 74.42%) were diagnosed as early-stage (FIGO IA–IIIB). In terms of gene analysis, only 6.67% (1/15) displayed a positive BRCA mutation. CA125 assay was performed in 71 patients, with a median CA125 level of 601.48 IU/mL (range 9.16–9035 IU/mL). Of these 71 patients, 17 (19.77%) had a normal CA125 level(mean = 20.21 ± 7.72 U/mL). More than half of patients assayed had a normal CA19–9(mean = 12.96 ± 9.01 U/mL) and/or HE4 value(mean = 65.86 ± 35.70pM) (36/66, 54.55%; 45/59, 76.27%) respectively. Co-existing endometriosis was found in 16 patients (18.60%) and 8 patients (9.30%) had a preoperative history or postoperative complications of VTE (Table 2).

**Table 2:** Characteristic of patients with developed venous thromboembolism (VTE)

Case	Age	FIGO stage	Ascites	SCS	R0/R1/RX	D-D ^a^	D-D ^b^	CA125	CA19–9	Time	DVT	PE	Survival	OS
1	55.00	IIIC	3000	2	R0	1.36	15.32	1664	96,649	Pre-operation	Left lower extremity		Alive	9
2	42.00	IIIC	2000	4	R0	12.86	17.42	446.2	35.78	Pre-operation	–	Yes	Alive	24
3	45.00	IC	200	4	R0	/	13.68	2612	10.72	Pre-operation	Right lower extremity		Alive	75
4	40.00	IIIC	200	2	R1	8.77	40	239.3	251.7	Pre-operation	Both lower extremity	Yes	Alive	24
5	70.00	IV	0	1	RX	2.43	5.25	911.5	1469	Post-operation	–	Yes	Alive	10
6	25.00	IV	2000	1	R0	12.48	5.69	9035	2.96	Post-operation	Both lower extremity		NA	–
7	66.00	IA	0	5	R0	0.78	6.15	9.5	10.54	Post-operation	Both lower extremity	Yes	Alive	9
8	56.00	IA	0	3	R0	/	/	103.1	8.42	Post-operation	Both lower extremity		Alive	13

*SCS* Surgical complexity score

*DVT* Deep venous thrombosis

*PE* Pulmonary embolism

*OS* Overall survival

^a^Preoperative d-dimer value

^b^Postoperative d-dimer value

### Treatments of the patients with OCCC

Complete staging surgery was performed in 64 (74.42%) patients and cytoreductive surgery was performed in 22 (25.58%) patients. Data related to the surgical procedures are summarized in Table 3. Lymphadenectomy was performed in 66 women (76.74%), and three of them (4.55%) were found to have positive LN. Of the 2225 LN resected, 2.07% (46/2225) were found to have metastatic lesions. 85.94% (55/64) patients at early stage had over 20 lymph nodes removed), whereas 77.27% (17/22) patients at advanced stage had less than 20 lymph nodes removed. Among patients with advanced OCCC, optimal cytoreduction was achieved in 77.27% (17/22) patients. Of the 5 patients that underwent suboptimal cytoreductive surgery, 1 patient’s family refused to remove the involved bowel, and 4 patients had multiple metastatic lesions. The mean length of hospital stays for patients undergoing staging surgery was 11.44 ± 6.45 days following completion of initial postoperative chemotherapy; and the mean length of hospital stays for those undergoing cytoreductive surgery was 15.00 ± 6.50 days following completion of initial postoperative chemotherapy.

**Table 3:** Surgical procedures and clinical outcomes

	Total	%	FIGO stage			
			I ~ IIIb(*n* = 64)	%	IIIc~IV(*n* = 22)	%
**Residual disease**						
No gross residual	77	89.53	64	100	13	59.09
0.1 ~ 1.0 cm	4	4.65	0	0	4	18.18
> 1 cm	5	5.81	0	0	5	22.73
**Ascites (ml)**						
< 500	69	80.23	59	92.19	10	45.45
500 ~ 1999	7	8.14	3	4.69	4	18.18
2000 ~ 4999	5	5.82	0	0	5	22.73
≥ 5000	3	3.49	0	0	3	13.64
NA	2	2.33	2	3.13	0	
**Number of lymph node resected**						
0	20	23.26	5	7.81	15	68.18
1 ~ 20	6	6.98	4	6.25	2	9.09
21 ~ 40	45	52.33	41	64.06	4	18.18
≥ 40	15	17.44	14	21.88	1	4.55
**Lymph node ratio (LNR)**^**a**^	46/2225	2.07	45/2034	2.21	1/191	0.52
**Estimated blood loss (ml)**						
< 100	4	4.65	3	4.69	1	4.55
100 ~ 499	46	53.49	38	59.38	8	36.36
500 ~ 999	26	30.23	19	29.69	7	31.82
≥ 1000	9	10.47	3	4.69	6	27.27
NA	1	1.16	1	1.56	0	0
**Operation type**						
Staging surgery	64	74.42	64	100	0	0
Standard cytoreduction	13	15.12	0	0	13	59.09
Radical cytoreduction	1	1.16	0	0	1	4.55
Extral-radical cytoreduction	3	3.49	0	0	3	13.64
Palliative surgery	5	5.81	0	0	5	22.73
**Surgical complexity score (SCS)**						
1 ~ 3	26	30.23	14	21.88	12	54.55
4 ~ 7	59	68.60	50	78.13	9	40.91
≥ 8	1	1.16	0	0	1	4.55
**Interval of initial postoperative chemotherapy (days)**	16.76 ± 8.01(6 ~ 39)		17.86 ± 7.94(6 ~ 39)		13.47 ± 7.28(6 ~ 32)	
**Length of hospital stays (days)**	12.15 ± 6.54(5 ~ 37)		11.44 ± 6.45(5 ~ 37)		15.00 ± 6.50(8 ~ 35)	
**Recurrence**	13	15.12	6	9.38	7	31.82
**Endpoint status**						
Alive	62	72.09	52	81.25	10	45.45
Cancer specific deaths	10	11.63	2	3.13	8	36.36
Loss to follow-up	14	16.28	10	15.63	4	18.18

^a^Lymph node ratio (LNR), defined as the ratio of the number of metastatic lymph nodes (MLNs) to the number of resected lymph nodes (RLNs)

Adjuvant chemotherapy was administered in our hospital in 76 patients (88.37%), with a combination of paclitaxel and platinum following surgery. The other 10 patients were discharged and attended a local hospital for their adjuvant chemotherapy. Among the 76 patients who received chemotherapy in our hospital, 36 (47.37%) completed the initial chemotherapy within 2 weeks of surgery and 68 (89.47%) within 4 weeks of surgery. The remaining 8 patients did not complete chemotherapy within 4 weeks due to anemia, infection, or personal reasons. The mean interval between surgery and initial postoperative chemotherapy was 16.76 ± 8.01 days (range 6–39 days).

### Clinical characteristics of OCCC patients with co-existing endometriosis

There were 16 patients found to have co-existing endometriosis. A comparison of the clinical characteristics of patients with and without co-existing endometriosis is presented in sTable1. Patients with co-existing endometriosis had a mean age of 45.50 ± 6.19 years old, and 68.75%of them were under 50 years old. Patients with endometriosis tended to be younger than those without endometriosis but without statistical significance (*P* = 0.078).

### Pathological characteristics

The immunohistochemical results are presented in Table 4. Most (26/31, 83.87%) patients tested positive for HNF1β and 91.67% (55/60), 71.93% (41/57), and 82.00% (41/50) of patients tested negative for WT-1, ER, and PR respectively. Of the 20 patients who were tested containing HNF1β, WT-1, ER, and PR, 11 (55%) had the combination of positive HNF1β, and negative WT-1, ER, and PR characteristics for the diagnosis of clear cell carcinoma. Of these 20 patients, 19 were alive and one was lost to follow-up. In addition, Ki-67 was positive in 1–20% of cells in 18/69 (26.09%) cases; 21–40% of cells in 26/69 (37.68%) cases; 41–60% of cells in 17/69 (24.64%) cases; 61–80% of cells in 7/69 (10.14%) cases; and > 80% of cells in 1/69 (1.45%) cases.

**Table 4:** Immunohistochemical characteristics of patients

	Total number^a^	Positive (%)	Negative (%)	Weak/partial positive (%)
HNF1β	31	26/31(83.87%)	1/31(3.23%)	4/31(12.90%)
WT-1	60	2/60(3.33%)	55/60(91.67%)	3/60(5.00%)
ER	57	3/57(5.26%)	41/57(71.93%)	13/57(22.81%)
PR	50	1/50(2.00%)	41/50(82.00%)	8/50(16.00%)
NapsinA	60	29/60(48.33%)	22/60(36.67%)	9/60(15.00%)
CK7	66	61/66(92.42%)	3/66(4.55%)	2/66(3.03%)
P53	54	16/54(29.63%)	16/54(29.63%)	22/54(40.74%)
P16	25	13/25(52.00%)	4/25(16.00%)	8/25(32.00%)
PAX8	41	40/41(97.56%)	1/41(2.44%)	0
CK20	34	0	32/34(94.12%)	2/34(5.88%)
CD10	18	1/18(5.56%)	14/18(77.78%)	3/18(16.67%)
CD15	47	24/47(51.06%)	11/47(23.40%)	12/47(25.53%)
Vim	38	7/38(18.42%)	28/38(73.68%)	3/38(7.89%)
CA125	37	26/37(70.27%)	4/37(10.81%)	7/37(18.92%)

^a^Number of patients who had this test

### Survival analysis of the clinical data

The median follow-up of the cohort (86 patients) was 53 months based on the reverse Kaplan-Meier method. Among the cohort, 14 patients experienced relapse shown in sTable2. After recurrence, five cases were performed with surgery and received chemotherapy, three of them are still alive, and the median post-relapse survival is 13 months; while eight cases were only performed with chemotherapy after recurrence and two cases are still alive, and the median post-relapse survival is 11 months. Among the seven patients having complete information about the chemotherapy after recurrence, one patient received ifosfamide + etoposide, and all the other six patients received taxanes ± platinum for relapse management. And the median post-relapse survival of these 14 patients is 13 months based on Kaplan-Meier analysis.

There were 71(81.61%) patients follow-up continuously. Among the 71 patients followed up, 49 were followed up for more than 1 year, 28 for more than 3 years, and only 7 for more than 5 years. Survival analysis was conducted among the 49 patients followed up for more than 1 year. Recurrence occurred in 13 patients (26.53%), including 7 cases at an advanced stage and 6 cases at an early stage. For patients with early and advanced stage OCCC, 1-year PFS rates were 94.59 and 33.33% respectively and 3-year PFS rates were 78.95 and 22.22% respectively; OS rates at 1-year were 97.30 and 66.67% respectively and at 3-years were 89.47 and 44.44% respectively. We also compared the median survival time of patients with IC1 or IC2/3 of OCCC, and the median PFS and OS of them are shown in sTable3 and sTable4. Although the statistical significance was not calculated due to the small number of cases, it can be clearly found that the prognosis of patients at stage IC1 (49 months) is better than that at IC2/3 (37.6 months).

Analysis of demographic and clinicopathological parameters associated with OS and PFS is presented in Table 5. Univariate analysis demonstrated that CA19–9 ≥ 70.3 U/mL (*P* = 0.038), advanced stage (*P* = 0.001) and ascites ≥2000 mL (*P* = 0.015) were significantly associated with reduced OS. Besides those factors, suboptimal cytoreduction(*P* = 0.085) was also included in multivariate analysis. Multivariate analysis revealed that CA19–9 ≥ 70.3 U/mL (*P* = 0.025) was an independent prognostic indicator of OS. Bootstrap analysis found ascites ≥2000 mL (*P* = 0.001) also had statistical significance.

**Table 5:** Univariate and multivariate analysis of factors associated with overall survival and progression free survival (*n* = 49)

	Overall survival					Progression free survival				
	Univariate analysis		Multivariate analysis			Univariate analysis		Multivariate analysis		
	OR (95% CI)	*P* value	OR (95% CI)	*P* value	Bootstrap *P* value ^a^	OR (95% CI)	*P* value	OR (95% CI)	*P* value	Bootstrap *P* value ^a^
Age	2.615(0.693 ~ 9.865)	0.156				3.274(1.069 ~ 10.030)	**0.038**	1.149(0.111 ~ 11.872)	0.907	0.736
ASA (I + II vs. III + IV)	0.204(0.025 ~ 1.668)	0.138				0.411(0.113 ~ 1.504)	0.179			
CA125	33.280(0.040 ~ 27,705.853)	0.307				25.129(0.008 ~ 79,761.009)	0.433			
CA19–9	9.707(1.134 ~ 83.096)	**0.038**	19.599(1.441 ~ 266.656)	**0.025**	0.104	3.930(0.933 ~ 16.554)	0.062	6.122(0.737 ~ 50.873)	0.094	0.052
HE4	64.472(0.089 ~ 46,669.453)	0.215				5.014(0.959 ~ 26.217)	0.056	9.898(0.962 ~ 101.809)	0.054	**0.027**
FIGO stage (IA ~ IIIB vs. IIIC~IV)	12.800(2.654 ~ 61.724)	**0.001**	1.637(0.145 ~ 18.447)	0.690	0.338	5.835(1.909 ~ 17.838)	**0.002**	1.369(0.130 ~ 14.396)	0.794	0.331
Ascites (≥2000 ml)	8.150(1.508 ~ 44.054)	**0.015**	20.924(0.940 ~ 465.542)	0.055	**0.001**	3.746(0.787 ~ 17.841)	0.097	18.498(0.964 ~ 354.767)	0.053	**0.001**
Lymphadenectomy	0.459(0.115 ~ 1.837)	0.271				0.448(0.137 ~ 1.466)	0.184			
Comorbid illnesses	0.755(0.188 ~ 3.026)	0.691				0.893(0.292 ~ 2.730)	0.842			
Multiple comorbid illnesses	1.263(0.155 ~ 10.301)	0.827				0.679(0.088 ~ 5.228)	0.710			
Thrombosis	0.042(0.000 ~ 911.409)	0.533				0.044(0.000 ~ 329.984)	0.492			
Endometriosis	0.549(0.069 ~ 4.402)	0.573				0.340(0.044 ~ 2.619)	0.300			
Interval ^b^ (> 14 days)	0.419(0.104 ~ 1.686)	0.221				0.440(0.143 ~ 1.349)	0.151			
Suboptimal cytoreduction	4.112(0.821 ~ 20.583)	0.085	1.026(0.064 ~ 16.408)	0.986	0.715	3.116(0.687 ~ 14.130)	0.141			
SCS (< 4 vs. ≥4)	0.523(0.140 ~ 1.959)	0.336				0.725(0.237 ~ 2.218)	0.573			

*ASA* American Society of Anesthesiologists class, *SCS* Surgical complexity score

^a^Based on 5000 bootstrap samples

^b^Interval. The interval of initial postoperative chemotherapy (days)

In univariate analyses of PFS, age ≥ 58 years (*P* = 0.038) and advanced stage (*P* = 0.002) were significantly associated with reduced PFS. Age ≥ 58 years and advanced stage, along with CA19–9 ≥ 70.3 U/mL, HE4 ≥ 94.5pM, and ascites ≥2000 mL were included in multivariate analysis. None of these factors had statistical significance, however, the additional bootstrap analysis found that HE4 ≥ 94.5pM (*P* = 0.027) and ascites ≥2000 mL (*P* = 0.001) were associated with a decreased PFS.

We have also calculated the power regarding the ability to detect significant differences of survival analysis and the multiple hypothesis correction using False Discovery Rate (FDR) approach for the indicators included in the multivariate analysis among these 49 patients, the power and adjusted *p* values were presented in sTable7 and sTable8. Unfortunately, none of the adjusted *p* values is statistically significant, which might be caused by the small samples of the patients in this study. Thus, a large-sample multicentral clinical research is urgently needed.

### Analysis of data from the SEER database

We analyzed the data of 179 patients enrolled from the SEER database, with an average age of 55.22 ± 10.47 (range 18–85) years. In terms of SEER summary stage, there were 55 cases of localized, 77 cases of regional and 47 cases of distant, while 40 patients were diagnosed at advanced stage (AJCC IIIC and IV). Lymphadenectomy was performed in 134 patients, of which 19 (14.18%) had positive LN. We also found that 6 patients had distant metastasis in bone (1 case), lung (1 case), and liver (4 cases).

**Fig. 2:**
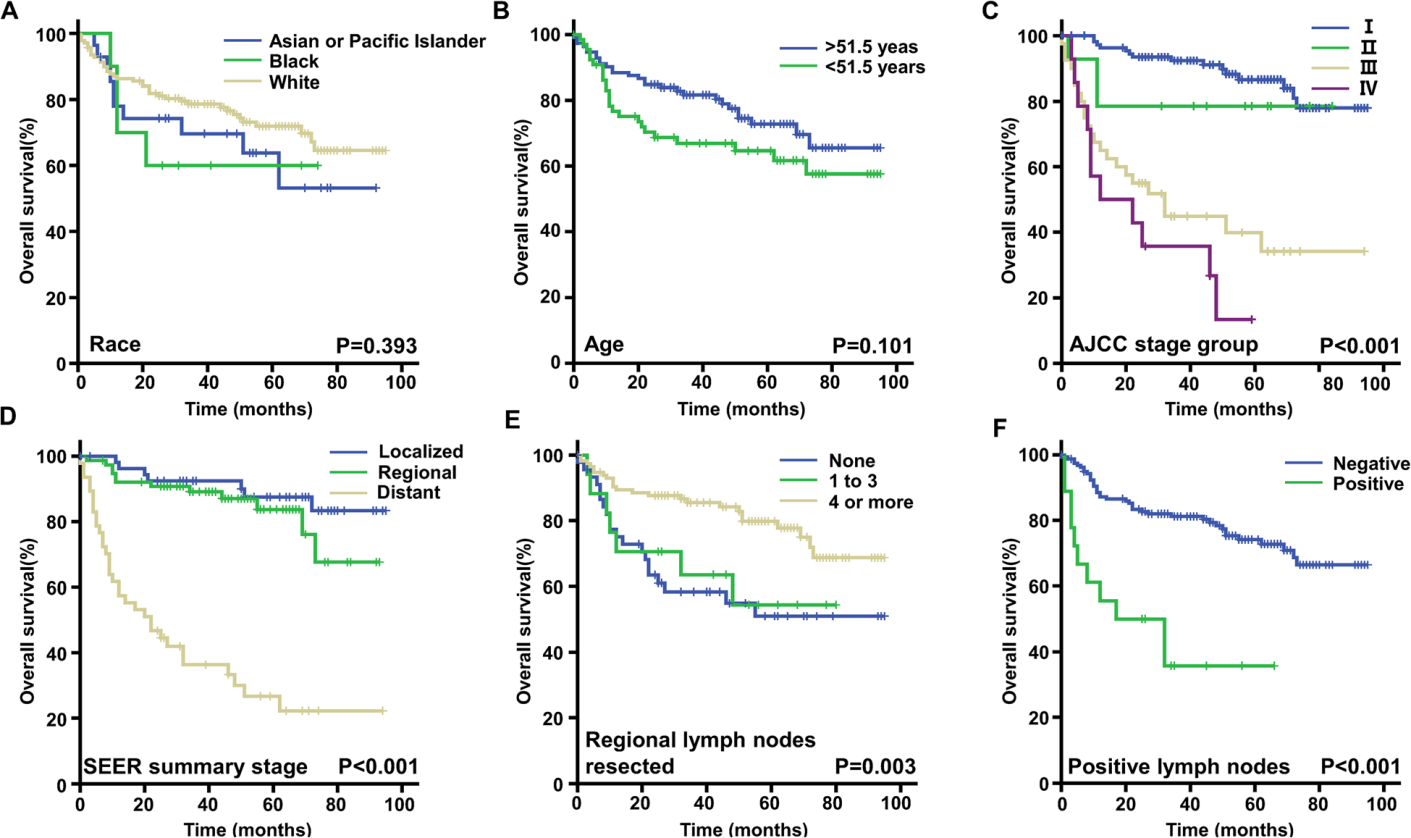
Kaplan-Meier curves for overall survival of OCCC patients obtained from SEER database. Grouped by race (**a**), age (**b**), AJCC stage group (**c**), SEER Summary stage (**d**), the number of regional lymph nodes resected (**e**), positive lymph nodes (**f**) for all the 179 patients obtained from SEER database

Survival analysis was performed on these 179 cases of OCCC with a 3-year OS rate of 56.98%. Kaplan-Meier survival curves (Fig. 2) were generated which showed that AJCC stage (*P* < 0.001), SEER summary stage (*P* < 0.001), ≥ 4 LN removed (*P* = 0.003), and positive LN (*P* < 0.001) were significantly associated OS in patients with OCCC. Multivariate analysis was performed with variables including AJCC stage, SEER summary stage, number of LN removed, and positive LN (Table 6). Patients with summary stage of distant had poorer survival (*P* = 0.011). Positive LN was an independent prognostic factor for the survival of OCCC (*P* = 0.001). AJCC stage and number of LN removed had no significant impact on OS.

**Table 6:** Multivariate analysis of factors associated with overall survival of the data from SEER database(*n* = 179)

	Multivariate analysis		
	OR (95% CI)	***P*** value	***P*** value ^**a**^
**AJCC stage group**			
I	1		
II	1.880(0.186 ~ 18.983)	0.593	0.354
III	3.043(0.255 ~ 36.253)	0.378	0.214
IV	0.526(0.236 ~ 1.171)	0.116	0.119
**Summary stage**			
Localized	1		
Regional	1.361(0.489 ~ 3.787)	0.555	0.556
Distant	21.152(2.019 ~ 221.646)	**0.011**	**0.009**
**Regional lymph nodes removed**			
None	1		
1 to 3 regions	1.847(0.929 ~ 3.672)	0.080	0.103
4 or more regions	0.621(0.243 ~ 1.587)	0.319	0.370
**Positive lymph nodes**	5.531(2.102 ~ 14.553)	**0.001**	**0.002**

^a^Based on 5000 bootstrap samples

## Discussion

OCCC is a rare pathological type of EOC, and there is geographic variance in the prevalence of OCCC, being more common in Asia [3–5]. Prevalence also differs by race, being higher in Asians (11.1%) and lower in black, white, and other populations (3.1, 4.8, and 5.5%, respectively) [15]. However, among patients analyzed from the SEER database in our research, only 15.08% of the 179 cases were Asians, with 78.21% of cases being white; this is likely related to the racial differences in the USA population. In the present study, 74.42% patients were diagnosed at an early stage in a younger age (49.21 ± 9.91), consistent with previous studies, showing a distinct epidemiology of OCCC from HGSC, which is more frequently diagnosed at an advanced stage with a poor prognosis [8].

Endometriosis is a common disease in women of reproductive age, which is recognized as a precancerous lesion of OCCC and is associated with triple the risk of OCCC [7, 16], approximately 18–43% of women with OCCC have a history of endometriosis [17, 18]. Endometriotic lesions often have high expression of HNF1β and carry multiple somatic mutations, such as ARID1A and PIK3CA, which are thought to occur early in the malignant transformation of OCCC [16]. The risk of tumorigenesis in endometriosis is about 1% among premenopausal women and 1–2.5% among postmenopausal women [17, 19]. A study by Ye et al. demonstrated that patients with OCCC and concurrent endometriosis were on average 8 years younger than those without, and were more likely to present at early stage (78.5%) [20]. However, although patients with co-existing endometriosis tend to have better survival outcomes, endometriosis was not an independent predictor of survival [21]. In the present study, OCCC patients with endometriosis also tended to be diagnosed at a younger age (average age of 45.50 ± 6.19) compared to patients without endometriosis, however, this difference was not significant (*P* = 0.078). Unfortunately, due to the small sample of our research, survival analysis of OCCC patients with and without endometriosis could not be constructed.

Currently, there is no specific biomarker for OCCC, patients with OCCC usually present with a mild elevation of serum CA125 [9]. In the present study, 19.77% of the patients had a normal level of CA125, and 38.37% of the patients had a CA125 level of < 100 U/mL. Thus, there is a need to identify the novel diagnostic markers to improve the early diagnosis of OCCC. Chronic inflammation appears to have an effect on tumorigenesis and response to therapy, as well as affecting prognosis [22]. Several systemic inflammatory response (SIR) biomarkers have been investigated as potential biomarkers of OCCC, such as the neutrophil-to-lymphocyte ratio (NLR) and lymphocyte-to-monocyte ratio (LMR). Most OCCC patients diagnosed at an early stage showed complete response to initial treatment with decreased NLR levels, and NLR was found to increase to preoperative high levels when recurrence, reflecting inflammation caused by the tumor [23]. Low LMR has been shown to be associated with advanced-stage disease, LN metastases, platinum-resistance, and poor prognosis, suggesting a decreased level of peripheral lymphocytes results in weakened immune surveillance and poor response to chemotherapy [22]. In addition, several potential diagnostic novel biomarkers were gradually discovered and used for OCCC, such as HNF1β, which is expressed in almost all cases of OCCC and now is used to distinguish histological subtypes by immunohistochemistry (IHC) [24]. OCCC lesions tend to be positive for CK7 and negative for CK20, ER, PR, WT-1, and p53 [10, 25]. Testing negative for α-fetoprotein and CD10 can be used to exclude yolk cell tumors and renal cell carcinoma [26]. Since there are few reports about combining those biomarkers, we analyzed immunohistochemical results of our patients, and 55% (11/20) of them had positive HNF1β and negative WT-1, ER, and PR. However, there remains an urgent need to discover novel biomarkers in peripheral blood or body fluids and validate their efficacy in the diagnosis of OCCC.

VTE, containing deep vein thrombosis (DVT) and pulmonary embolism (PE), are common in patients with OCCC, with a 2.5–4 times higher risk than in other subtypes of EOC [20, 27]. VTE is more commonly seen in advanced stage OCCC (21.9%) compared with early-stage (8.2%) disease, and occurred most commonly prior to primary surgery (36.4%), or with recurrence or progression (33.3%) [20]. There are some measurable biomarkers of increased risk of VTE in ovarian cancer, such as elevated platelet count, white blood cell counts, d-dimer and CA125 level, decreased hemoglobin and albumin levels preoperatively, and elevated d-dimer and decreased albumin postoperatively [28]. In our study, 8 patients developed VTE (Table 2), 4 preoperatively and 4 postoperatively. The low occurrence of VTE in our research might be associated with the use of appropriate prophylaxis. In spite of prophylaxis, patients with OCCC can still develop VTE, suggesting that an aggressive postoperative anticoagulation regimen and prolonged post-discharge VTE prophylaxis should be considered for patients with OCCC.

Staging surgery or optimal cytoreduction combined with chemotherapy is a common therapeutic strategy recommended for OCCC, however, only 11–27% of patients with OCCC respond to conventional platinum-based chemotherapy, resulting in a poor prognosis [29, 30]. Disorder of the cell’s detoxification effects of the glutathione system, low proliferation activity of OCCC, and overexpression of EGFR, HNF1β, and HER2 may be involved in chemoresistance [31]. In our clinical practice, OCCC patients often developed resistance characterized by a slow decline or elevation of tumor markers during postoperative chemotherapy. However, in the absence of more effective treatments, platinum-based chemotherapy remains the first-line adjuvant therapy for OCCC patients, and more effective therapies are urgently needed. Recently, several targeted therapies and immunotherapies have been investigated for use in OCCC, such as PARP, EZH2, and ATR inhibitors combined with synthetic lethality of ARID1A-deficiency, and MAPK/PI3K/HER2, VEGF/bFGF/PDGF, HNF1β, and PD-1/PD-L1 inhibitors. Some regimens have demonstrated efficacy and revealed a potential therapeutic benefit for OCCC patients, but further research is required [32–34]. We have collected all cases diagnosed with OCCC, and found that almost all of them were received surgery firstly, only one patient received NACT prior to debulking and she is still alive, this patient was not included in our data. We suspect the low number of NACT in patients with OCCC may be the cause of the following point: OCCC patients tend to be younger when they were firstly diagnosed without so many complications as the elders, which means they can withstand the extensive optimal cytoreduction. The same situation was also found in two clinical trials, CHORUS and EORTC55971 which compared the outcomes of patients with advanced EOC who had primary cytoreduction and NACT (neoadjuvant chemotherapy) + interval cytoreduction, and the incidence of OCCC was low (1.5 to 6.0%) in both trials [35, 36]. Therefore, the importance of NACT for OCCC requires multi-center and large-sample clinical studies.

Survival analysis of our clinical data identified levels of CA19–9 and ascites(≥2000 ml) as independent OS related factors in OCCC; HE4 and ascites(≥2000 ml), as independent PFS related factors in OCCC, have rarely been reported in previous studies. Elevated postoperative CA19–9 has been reported as an independent risk factor for reduced survival outcomes in OCCC patients with normal postoperative CA125 levels [37]. HE4 is not commonly used in OCCC prediction, however, Mckinnon found that since HE4 is sensitive to hormonal treatment and menstrual cycle variation, it may be potentially superior to CA125 as an endometriosis marker and therefore has potential as a marker for the risk of developing ovarian cancer [38]. Suboptimal cytoreduction and advanced stage have been found to be associated with less favorable survival outcomes in univariate analysis, however, neither is an independent prognostic factor in the present study, which may be related to the small sample size. The extent of lymph node dissection in patients with early OCCC has always been a controversial topic. In our survival analysis of 179 cases of OCCC from the SEER, we found that positive LN is an independent prognostic factor. OCCC tends to metastasize most frequently via the lymphatic system [39]. However, among patients in our hospital, only 4.48% of patients had metastatic LNs, which may be due to the fact that most (89.55%) of the 67 patients who received lymphadenectomy were at an early stage. Mueller found that 4.4–20% of clinically stage I OCCC had lymph node involvement, and this rate will be higher with positive cytology or ovarian surface involvement, accounting for 37.5% of metastases [40]. Therefore, we suggested that systematic pelvic and para-aortic lymphadenectomy is vital to accurately define the stage, provide prognostic information, and guide adjuvant therapy. Since ascites were mostly in advanced- stage disease, and cytoreduction was only used for advanced-stage of OCCC, we have also conducted a stratified multivariate analysis in early-stage and advanced-stage disease, respectively. However, according to the results presented in sTable5 and sTable6, CA19–9, HE4, and multiple comorbid illnesses are related to OS of patients with OCCC at early stage; lymphadenectomy and SCS ≥ 4 are related to OS of patients with OCCC at advanced stage in univariate analysis respectively, while only a high ASA score is a significant indicator for a poor PFS of patients with advanced OCCC in multivariate analysis.

However, this is a retrospective research in a single-center, our study still has some limitations. Due to the small sample of our study, we also analyzed the data from 179 patients enrolled from the SEER database, which used the different stage systems, including SEER summary stages as well as AJCC stage systems. Similar to us, most patients in this database were diagnosed at early stage (77.65%). In our study, there are only 3(4.48%) patients were found to have positive LN, we cannot know the statistical relationship between positive lymph nodes and prognosis. However, in the SEER database, lymphadenectomy was performed in 134 patients. 14.18% patients had positive LN, and positive LN was significantly associated with OS, which has made up for the limitations of our research. However, we still cannot find the most of the key findings of this study such as the HE4 and CA19–9 which were not available in SEER data. Therefore, multi-center clinical research with large samples is very necessary to investigate this rare disease.

In conclusion, our study presents the clinicopathological features, treatment regimens, and prognosis of OCCC in China, and confirms that OCCC typically presents at an early stage and at a younger age, with a mild elevation in CA125 level. Positive HNF1β, and negative WT-1, ER, and PR are reliable immunohistochemical indicators of OCCC. Patients with early-stage OCCC tend to have a better OS and PFS, and CA19–9, HE4, massive ascites, and positive lymph nodes are independent prognostic indicators. The present study confirms the unique features of OCCC, and further research is required to illustrate the molecular mechanisms, discover novel diagnostic biomarkers and targeted therapies, in order to contribute to the early diagnosis and better prognosis of OCCC.

## Supplementary Information

**Additional file 1: sTable1**. Clinical characteristics of patients with/without endometriosis. a. Mean ± standard deviation, range; NA. Not available. **sTable2**. The treatment and survival outcomes of the 14 patients with OCCC who had a recurrence. **sTable3**. The prognostic outcomes of patients with OCCC at the stage of IC. **sTable4**. The comparison of survival time between patients with OCCC at stage IC1 and IC2/3. **sTable5**. Multivariate analysis of factors associated with overall survival among patients at early stage (*n* = 37). a. Based on 5000 bootstrap samples. b. Interval. The interval of initial postoperative chemotherapy (days). ASA: American Society of Anesthesiologists class; SCS: Surgical complexity score. **sTable6**. Multivariate analysis of factors associated with overall survival among patients at advanced stage (*n* = 12). a. Based on 5000 bootstrap samples. b. Interval. The interval of initial postoperative chemotherapy (days). ASA: American Society of Anesthesiologists class; SCS: Surgical complexity score. **sTable7**. Power calculation and multiple hypothesis correction of the analysis of overall survival among the 49 patients with OCCC at early and advanced stage. **sTable8**. Power calculation and multiple hypothesis correction of the analysis of progression free survival among the 49 patients with OCCC at early and advanced stage.

## Data Availability

The datasets used and/or analyzed during the current study are available from the corresponding author on reasonable request.

## Abbreviations

EOC: Epithelial ovarian carcinoma
OCCC: Ovarian clear cell carcinoma
SEER: Surveillance, Epidemiology and End Results
VTE: Venous thromboembolism
OS: Overall survival
PFS: Progression-free survival
HGSC: High-grade serous carcinoma
HNF1β: hepatocyte nuclear factor 1β
ER: Estrogen receptor
PR: Progesterone receptor
WT-1: Wilms Tumor 1
PIK3CA: Phosphatidylinositol-4,5-bisphosphate 3-kinase catalytic subunit alpha
ARID1A: AT-rich interactive domain-containing protein 1A
LN: Lymph node
USTC: The First Affiliated Hospital of University of Science and Technology of China
CT: Computed tomography
MRI: Magnetic resonance imaging
PET-CT: Positron emission tomography-CT
BMI: Body mass index
ASA: American Society of Anesthesiologists class
SCS: Surgical complexity score
ELICA: Electrochemiluminescence immunoassay
FIGO: International Federation of Gynecology and Obstetrics
WHO: World Health Organization
AJCC: American Joint Committee on Cancer
ROC: Receiver operating characteristic
SIR: Systemic inflammatory response
NLR: Neutrophil-to-lymphocyte ratio
LMR: Lymphocyte-to-monocyte ratio
IHC: Immunohistochemistry
DVT: Deep vein thrombosis
PE: Pulmonary embolism
