# Supplementary material for: Clinical characteristics and prognosis of ovarian clear cell carcinoma: a 10-year retrospective study
Source: BMC Cancer. 2021 Mar 25;21:322. doi: 10.1186/s12885-021-08061-7 (PMC7993454; doi:10.1186/s12885-021-08061-7)
Supplement: Supplementary file 1 — Additional file 1: sTable1. Clinical characteristics of patients with/without endometriosis. a. Mean ± standard deviation, range; NA. Not available. sTable2. The treatment and survival outcomes of the 14 patients with OCCC who had a recurrence. sTable3. The prognostic outcomes of patients with OCCC at the stage of IC. sTable4. The comparison of survival time between patients with OCCC at stage IC1 and IC2/3. sTable5. Multivariate analysis of factors associated with overall survival among patients at early stage (n = 37). a. Based on 5000 bootstrap samples. b. Interval. The interval of initial postoperative chemotherapy (days). ASA: American Society of Anesthesiologists class; SCS: Surgical complexity score. sTable6. Multivariate analysis of factors associated with overall survival among patients at advanced stage (n = 12). a. Based on 5000 bootstrap samples. b. Interval. The interval of initial postoperative chemotherapy (days). ASA: American Society of Anesthesiologists class; SCS: Surgical complexity score. sTable7. Power calculation and multiple hypothesis correction of the analysis of overall survival among the 49 patients with OCCC at early and advanced stage. sTable8. Power calculation and multiple hypothesis correction of the analysis of progression free survival among the 49 patients with OCCC at early and advanced stage. [file 12885_2021_8061_MOESM1_ESM.docx]

**sTable1. Clinical characteristics of patients with/without endometriosis.**

|  | | With (n=16) | | Without (n=70) | | P value |
| --- | --- | --- | --- | --- | --- | --- |
|  | | No. | % | No. | % |  |
| Age (years)^a^ | | 45.50±6.19(35~54) |  | 50.09±10.40(25~70) |  | 0.083 |
|  | <40 | 3 | 18.75 | 12 | 17.14 |  |
|  | 40~49 | 8 | 50.00 | 19 | 27.14 |  |
|  | 50~59 | 5 | 31.25 | 28 | 40.00 |  |
|  | ≥60 | 0 | 0 | 11 | 15.71 |  |
| FIGO stage | |  |  |  |  | 0.312 |
|  | ⅠA~ⅢB | 14 | 87.50 | 50 | 71.43 |  |
|  | ⅢC~Ⅳ | 2 | 12.50 | 20 | 28.57 |  |
| Synchronous endometrial cancer | | 0 | 0 | 3 | 4.29 | 1.000 |
| Preoperative laboratory test | |  |  |  |  |  |
|  | CA125(U/ml) ^a^ | 117.17±126.84(9.81~446.20) |  | 682.20±1656.07(9.16~9035.00) |  | 0.292 |
|  | Normal (<35) | 3 | 18.75 | 14 | 20.00 |  |
|  | 35~99 | 3 | 18.75 | 13 | 18.57 |  |
|  | 100~499 | 4 | 25.00 | 19 | 27.14 |  |
|  | 500~999 | 0 | 0 | 4 | 5.71 |  |
|  | ≥1000 | 0 | 0 | 10 | 14.29 |  |
|  | NA | 6 | 37.50 | 10 | 14.29 |  |
|  | CA199(U/ml) ^a^ | 67.31±70.10(15.14~251.70) |  | 1818.43±12788.67 (0.60~96649.00) |  | 0.690 |
|  | Normal (<37) | 4 | 25.00 | 32 | 45.71 |  |
|  | 37~99 | 4 | 25.00 | 9 | 12.86 |  |
|  | 100~499 | 1 | 6.25 | 13 | 18.57 |  |
|  | ≥500 | 0 | 0 | 2 | 2.86 |  |
|  | NA | 7 | 43.75 | 14 | 20.00 |  |
|  | HE4 (pM) ^a^ | 73.01±63.86(21.00~228.00) |  | 111.31±107.53(14.00~669.00) |  | 0.313 |
|  | Normal (<140) | 8 | 50.00 | 37 | 52.86 |  |
|  | 140~499 | 1 | 6.25 | 11 | 15.71 |  |
|  | ≥500 | 0 | 0 | 1 | 1.43 |  |
|  | NA | 7 | 43.75 | 21 | 30.00 |  |

1. Mean ± standard deviation, range; NA. Not available

**sTable2. The treatment and survival outcomes of the 14 patients with OCCC who had a recurrence.**

| Case | Initial stage | Surgery | Residual disease | Cycle of post-operative chemotherapy | Management after relapse | Chemotherapy regimen after relapse |  | Status after relapse | Post-relapse survival |
| --- | --- | --- | --- | --- | --- | --- | --- | --- | --- |
| 1 | IIIC | Primary Cytoreductive surgery | R0 | 6 | Chemotherapy | Not available |  | Alive | 11 |
| 2 | IIB | Staging surgery | R0 | 6 | Surgery + chemotherapy | Paclitaxel + carboplatin |  | Alive | 10 |
| 3 | IIIC | Primary Cytoreductive surgery | R1 | 8 | Chemotherapy | Docetaxel + cisplatin;  Docetaxel + etoposide; |  | Dead | 6 |
| 4 | IIIC | Primary Cytoreductive surgery | RX | 6 | Chemotherapy | Not available |  | Dead | 32 |
| 5 | IC1 | Staging surgery | R0 | 6 | Palliative therapy | No chemotherapy |  | Dead | 0 |
| 6 | IIIC | Primary Cytoreductive surgery | R0 | 6 | Chemotherapy | Not available |  | Dead | 3 |
| 7 | IC3 | Staging surgery | R0 | 6 | Surgery + chemotherapy | Docetaxel(weekly);  Docetaxel + lobaplatin;  Liposomal doxorubicin; |  | Dead | 6 |
| 8 | IV | Primary Cytoreductive surgery | RX | 4 | Surgery + Chemotherapy | Docetaxel + oxaliplatin;  Gemcitabine+ oxaliplatin;  Etoposide+ lobaplatin;  Paclitaxel + lobaplatin;  Gemcitabine+ nedaplatin; |  | Dead | 13 |
| 9 | IC1 | Staging surgery | R0 | 3 | Surgery | No chemotherapy |  | Alive | 3 |
| 10 | IIIC | Primary Cytoreductive surgery | R0 | 7 | Chemotherapy | Not available |  | Dead | 11 |
| 11 | IIIC | Primary Cytoreductive surgery | R0 | 6 | Chemotherapy | Not available |  | Dead | 47 |
| 12 | IIIC | Unilateral ovariosalpingectomy+ Cytoreductive surgery | R0 | 3 | Chemotherapy | Ifosfamide + etoposide; |  | Dead | 2 |
| 13 | IC | Unilateral ovariosalpingectomy+ Staging surgery | R0 | 5 | Surgery + chemotherapy | Docetaxel + lobaplatin;  Paclitaxel + carboplatin;  Paclitaxel + lobaplatin;  Albumin-bound paclitaxel (weekly);  Gemcitabine+ carboplatin;  Gemcitabine+ oxaliplatin; |  | Alive | 27 |
| 14 | IC1 | Unilateral ovariosalpingectomy+ Staging surgery | R0 | 5 | Chemotherapy | Paclitaxel + lobaplatin;  Docetaxel + lobaplatin;  Docetaxel + nedaplatin; |  | Alive | 25 |

**sTable3. The prognostic outcomes of patients with OCCC at the stage of** Ⅰ**C.**

| Case | Stage | OS | Dead | PFS | Progression |
| --- | --- | --- | --- | --- | --- |
| 1 | ⅠC NA | 53 | 2 | 26 | Yes |
| 2 | ⅠC NA | 75 | 2 | 75 | No |
| 3 | ⅠC NA | 15 | 2 | 15 | No |
| 4 | ⅠC1 | 14 | 1 | 14 | Yes |
| 5 | ⅠC1 | 41 | 2 | 41 | No |
| 6 | ⅠC1 | 55 | 2 | 55 | No |
| 7 | ⅠC1 | 36 | 2 | 11 | Yes |
| 8 | ⅠC1 | 42 | 2 | 39 | Yes |
| 9 | ⅠC1 | 56 | 2 | 56 | No |
| 10 | ⅠC2 | 44 | 2 | 44 | No |
| 11 | ⅠC2 | 34 | 2 | 34 | No |
| 12 | ⅠC2 | 20 | 2 | 20 | No |
| 13 | ⅠC3 | 12 | 1 | 0 | Yes |
| 14 | ⅠC3 | 37 | 2 | 37 | No |

**sTable4. The comparison of survival time between patients with OCCC at stage** Ⅰ**C1 and** Ⅰ**C2/3.**

| Stage | PFS | | OS | |
| --- | --- | --- | --- | --- |
|  | **Median** | **95%CI** | **Median** | **95%CI** |
| ⅠC1 | 38.667 | 23.085~54.249 | 49.000 | 36.475~61.525 |
| ⅠC2/3 | 35.200 | 19.773~50.627 | 37.600 | 26.380~48.820 |

CI: Confidence interval

**sTable5. Multivariate analysis of factors associated with overall survival among patients at early stage (n=37)**

|  | Overall survival | | | | Progression free survival |
| --- | --- | --- | --- | --- | --- |
|  | Univariate analysis | Multivariate analysis | | | Univariate analysis |
|  | P value | OR (95% CI) | P value | Bootstrap P value ^a^ | P value |
| Age | 0.343 | / | / | / | 0.435 |
| ASA (Ⅰ+Ⅱ vs. Ⅲ+Ⅳ) | 0.243 | / | / | / | 0.891 |
| CA125 | 0.059 | 0.998(0.956~1.042) | 0.937 | 0.333 | 0.267 |
| CA199 | **0.019** | 1.023(0.841~1.244) | 0.819 | 0.145 | 0.389 |
| HE4 | **0.027** | 1.061(0.832~1.354) | 0.630 | 0.203 | 0.056 |
| Ascites (≥2000ml) | NA | / | / | / | NA |
| Lymphadenectomy | 0.591 | / | / | / | 0.992 |
| Comorbid illnesses | 0.727 | / | / | / | 0.588 |
| Multiple comorbid illnesses | **0.035** | NA | 0.453 | 0.103 | 0.572 |
| Thrombosis | 0.767 | / | / | / | 0.569 |
| Endometriosis | 0.283 | / | / | / | 0.844 |
| Interval ^b^ (>14days) |  | / | / | / |  |
| Suboptimal cytoreduction | NA | / | / | / |  |
| SCS (<4 vs. ≥4) |  | / | / | / |  |

a. Based on 5000 bootstrap samples.

b. Interval. The interval of initial postoperative chemotherapy(days)

ASA: American Society of Anesthesiologists class; SCS: Surgical complexity score

**sTable6. Multivariate analysis of factors associated with overall survival among patients at advanced stage (n=12)**

|  | Overall survival | | | | Progression free survival | | | |
| --- | --- | --- | --- | --- | --- | --- | --- | --- |
|  | Univariate analysis | Multivariate analysis | | | Univariate analysis | Multivariate analysis | | |
|  | P value | OR (95% CI) | P value | Bootstrap P value ^a^ | P value | OR (95% CI) | P value | Bootstrap P value ^a^ |
| Age | 0.456 | / | / | / | 0.166 | / | / | / |
| ASA (Ⅰ+Ⅱ vs. Ⅲ+Ⅳ) | 0.116 | / | / | / | **0.018** | 0.134(0.014~1.324) | 0.085 | **0.020** |
| CA125 | 0.337 | / | / | / | 0.391 | / | / | / |
| CA199 | 0.178 | / | / | / | 0.212 | / | / | / |
| HE4 | 0.411 | / | / | / | 0.190 | / | / | / |
| Ascites (≥2000ml) | 0.414 | / | / | / | 0.836 | / | / | / |
| Lymphadenectomy | **0.016** | NA | 0.947 | 0.579 | **0.087** | 2.469(0.502~12.158) | 0.266 | 0.074 |
| Comorbid illnesses | 0.342 | / | / | / | 0.190 | / | / | / |
| Multiple comorbid illnesses | 0.232 | / | / | / | 0.126 | / | / | / |
| Thrombosis | 0.232 | / | / | / | 0.126 | / | / | / |
| Endometriosis | 0.232 | / | / | / | 0.126 | / | / | / |
| Interval ^b^ (>14days) | 0.702 | / | / | / | 0.780 | / | / | / |
| Suboptimal cytoreduction | 0.948 | / | / | / | 0.959 | / | / | / |
| SCS (<4 vs. ≥4) | **0.037** | NA | 0.959 | 0.623 | 0.340 | / | / | / |

a. Based on 5000 bootstrap samples.

b. Interval. The interval of initial postoperative chemotherapy(days)

ASA: American Society of Anesthesiologists class; SCS: Surgical complexity score

**sTable7. Power calculation and multiple hypothesis correction of the analysis of overall survival among the 49 patients with OCCC at early and advanced stage.**

|  | Univariate analysis | | Multivariate analysis | | |
| --- | --- | --- | --- | --- | --- |
|  | Power | P value | P value | Adjusted p value | Bootstrap P value ^a^ |
| Age | 0.19 | 0.156 | / | / | / |
| ASA (Ⅰ+Ⅱ vs. Ⅲ+Ⅳ) | 1.00 | 0.138 | / | / | / |
| CA125 | 1.00 | 0.307 | / | / | / |
| CA19-9 | 0.64 | **0.038** | **0.025** | 0.100 | 0.104 |
| HE4 | 1.00 | 0.215 | / | / | / |
| FIGO stage (ⅠA~ⅢB vs. ⅢC~Ⅳ) | 0.99 | **0.001** | 0.690 | 0.920 | 0.338 |
| Ascites (≥2000ml) | 0.99 | **0.015** | 0.055 | 0.110 | **0.001** |
| Lymphadenectomy | 0.97 | 0.271 | / | / | / |
| Comorbid illnesses | 1.00 | 0.691 | / | / | / |
| Multiple comorbid illnesses | 1.00 | 0.827 | / | / | / |
| Thrombosis | 1.00 | 0.533 | / | / | / |
| Endometriosis | 1.00 | 0.573 | / | / | / |
| Interval ^b^ (>14days) | 1.00 | 0.221 | / | / | / |
| Suboptimal cytoreduction | 1.00 | 0.085 | 0.986 | 0.986 | 0.715 |
| SCS (<4 vs. ≥4) | 1.00 | 0.336 | / | / | / |

**sTable8. Power calculation and multiple hypothesis correction of the analysis of progression free survival among the 49 patients with OCCC at early and advanced stage.**

|  | Univariate analysis | | Multivariate analysis | | |
| --- | --- | --- | --- | --- | --- |
|  | Power | P value | P value | Adjusted p value | Bootstrap P value ^a^ |
| Age | 0.62 | **0.038** | 0.907 | 0.907 | 0.736 |
| ASA (Ⅰ+Ⅱ vs. Ⅲ+Ⅳ) | 0.99 | 0.179 | / | / | / |
| CA125 | 1.00 | 0.433 | / | / | / |
| CA19-9 | 0.99 | 0.062 | 0.094 | 0.157 | 0.052 |
| HE4 | 0.96 | 0.056 | 0.054 | 0.135 | **0.027** |
| FIGO stage (ⅠA~ⅢB vs. ⅢC~Ⅳ) | 1.00 | **0.002** | 0.794 | 0.993 | 0.331 |
| Ascites (≥2000ml) | 0.60 | 0.097 | 0.053 | 0.265 | **0.001** |
| Lymphadenectomy | 0.29 | 0.184 | / | / | / |
| Comorbid illnesses | 0.55 | 0.842 | / | / | / |
| Multiple comorbid illnesses | 1.00 | 0.710 | / | / | / |
| Thrombosis | 1.00 | 0.492 | / | / | / |
| Endometriosis | 1.00 | 0.300 | / | / | / |
| Interval ^b^ (>14days) | 0.68 | 0.151 | / | / | / |
| Suboptimal cytoreduction | 0.87 | 0.141 | / | / | / |
| SCS (<4 vs. ≥4) | 0.06 | 0.573 | / | / | / |
